# The Rapid Synthesis of Integral Stimuli

**DOI:** 10.1162/opmi_a_00208

**Published:** 2025-05-23

**Authors:** C. E. R. Edmunds, Fraser Milton, Andy J. Wills

**Affiliations:** School of Social Sciences, University of the West of England, Bristol, UK; School of Psychology, University of Exeter, Exeter, UK; School of Psychology, University of Plymouth, Plymouth, UK

**Keywords:** categorization, integral, separable, Combination Theory, Differentiation Theory, analytic, holistic

## Abstract

Integral stimuli (e.g., colors varying in saturation and brightness) are classically considered to be processed holistically (i.e., as undifferentiated stimulus wholes); people analyze such stimuli into their consistent dimensions only with substantial time, effort, training, or instruction (Foard & Kemler, 1984). In contrast, Combination Theory (Wills et al., 2015) argues that the dimensions of integral stimuli are quickly combined. Through an investigation of the effects of stimulus presentation time, we support Combination Theory over the classical holistic-to-analytic account. Specifically, using colored squares varying in saturation and brightness, we demonstrate that the prevalence of single-dimension classification increases as stimulus presentation time is reduced. We conclude that integral stimuli are not slowly analyzed, they are quickly synthesized.

## INTRODUCTION

Even the simplest of laboratory-based stimuli tend to vary across more than one stimulus attribute. Monochromatic squares are presented at different sizes and brightnesses (Smith & Kemler, 1984); pure sine waves at different pitches and intensities (Grau & Nelson, 1988). For many decades, there has been consensus that one of the ways in which multi-attribute stimuli differ from each other is in the level of separability of their dimensions (Garner, 1976). For highly separable stimulus dimensions, such as size and brightness, adults find it easy to attend to one of the stimulus dimensions while ignoring the other. In contrast, for integral stimulus dimensions such as pitch and loudness, selective attention is difficult (Garner, 1976). Nonetheless, the stimulus dimensions of integral stimuli have psychological reality, as shown by the fact that it is even harder to selectively attend along an arbitrary dimension in stimulus space. For example, for colored squares of a fixed hue but varying in saturation and brightness, it is easier to classify on the basis of saturation, or brightness, than it is to classify on the basis of arbitrary dimensions that are a 45-degree rotation of the saturation-brightness axes (Foard & Kemler, 1984). Thus, integral dimension pairs, such as saturation and brightness, are both difficult to selectively attend, and are considered as primary or ‘privileged’ stimulus dimensions.

The study of integral stimuli seems to have been key to the development of a class of theories of processing order in stimulus perception and classification (Lockhead, 1972 et seq.); this class of account being subsequently described as Differentiation Theory (Wills et al., 2015). Under Differentiation Theory, integral stimuli are initially perceived as undifferentiated wholes, or “blobs”. It proposes that if the task at hand cannot be completed with this holistic stimulus representation then, with time and effort, people can analyze the stimulus into its constituent dimensions.

The domain of Differentiation Theory was widened by subsequent investigators, who argued that even stimuli that were separable for adults under conditions of intentional unspeeded classification (e.g., grey squares varying in size and brightness) were classified holistically by children (Smith & Kemler, 1977), by adults under time pressure (Smith & Kemler, 1984; Ward, 1983) or cognitive load (Smith & Shapiro, 1989), and by adults who classified under incidental rather than intentional conditions (Kemler Nelson, 1984). Thus, Differentiation Theory was considered to apply quite broadly; people start with an undifferentiated stimulus whole, which they break down into its constituent components if they have the time and mental resources to do so. Under this account, separable and integral stimuli differ in the time or effort required to analyze the stimulus into its component dimensions; with integral and separable stimuli seen as the two poles of a continuum of analyzability.

The application of Differentiation Theory to separable stimuli turned out to be an over-extension made on the basis of flawed analyses. In correcting these flaws, Wills et al. (2015) demonstrated that reduced stimulus presentation time, cognitive load, and incidental training conditions, *increased* the likelihood that people would classify separable stimuli on the basis of a single stimulus dimension (rather than decrease it as predicted by Differentiation Theory). This pattern of results is predicted by a class of accounts starting with Neisser (1967), which were subsequently described as Combination Theory (Wills et al., 2015).

Combination Theory is the inverse of Differentiation Theory. It argues that cognition begins with the stimulus attributes (e.g., saturation, brightness), and that these attributes are combined if time and mental resources allow. It provides a sufficient account of the effects of time pressure (both in terms of stimulus presentation time and limited response time), cognitive load and incidental training on the classification of separable stimuli (Wills et al., 2015). However, it makes a striking and untested prediction concerning integral stimuli. It predicts than when the time available to process the stimuli is sufficiently low, this will increase the prevalence of single-dimension classification of integral stimuli. Thus, despite the difficulty people have in selectively attending to one dimension of an integral stimulus under unlimited stimulus presentation times, with sufficiently short presentations times their classification will nonetheless more likely be on the basis of a single stimulus dimension, because they have not yet combined the dimensions. In other words, the properties of integral stimuli under unlimited stimulus presentation times come not from the difficulty of differentiating the holistic ‘blob’ into its constituent dimensions (as Differentiation Theory would predict) but from the rapidity with which the stimulus dimensions are combined. In order to explain performance under conditions of unlimited stimulus presentation times, Combination Theory must further assume that, once combined, selective attention of dimensions is effortful. This is an existing assumption of Combination Theory, already employed to explain other phenomena (Wills et al., 2015).

In summary, Combination Theory predicts that reducing the stimulus presentation time will increase the prevalence of single-dimension classification of integral stimuli, while Differentiation Theory predicts the opposite, or the absence of an effect.

### The Current Experiments

**Figure 1.**
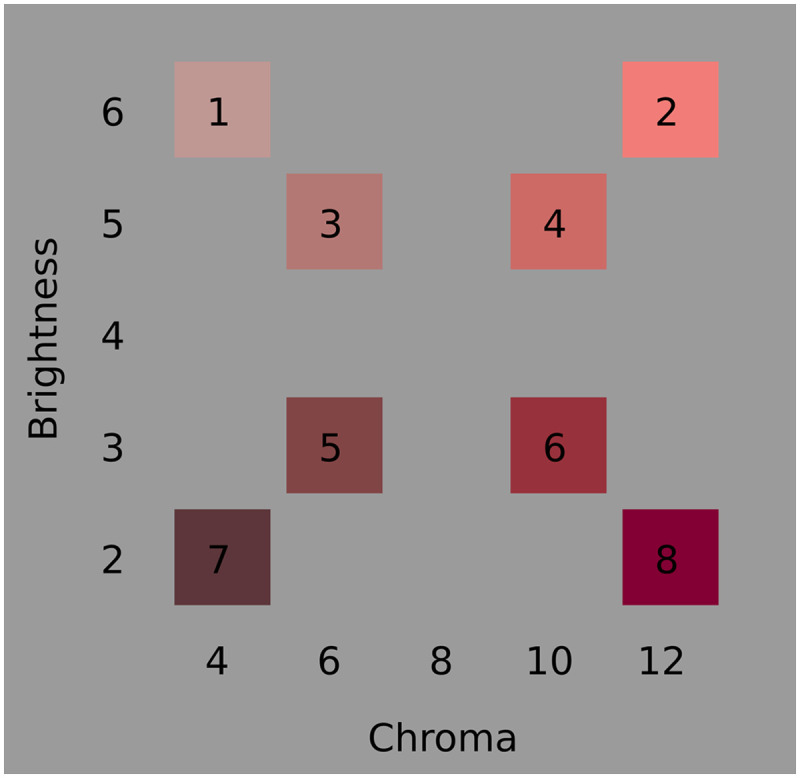
The eight stimuli employed in the current experiments, along with their Munsell Chroma and Brightness values. The text and numbers in this figure are for reference; only the coloured squares themselves were presented to participants.

A key experimental procedure employed in support of Differentiation Theory is the restricted classification (or “triad”) task (Garner, 1976; Smith & Kemler, 1984; Ward, 1983). Participants are presented with three stimuli, for example the stimuli labelled 6 to 8 in Figure 1. Stimuli 7 and 8 are identical in brightness but quite dissimilar in saturation (‘chroma’). Stimuli 6 and 8 are similar on both dimensions, but identical on neither. The task is to pick the two stimuli that ‘go together’ (or to pick the odd one out). No feedback is given.

A defining characteristic of integral stimuli is that people have a strong preference to group stimuli 6 and 8 together in this task, i.e., the stimuli that are similar, but not the same, on both dimensions. This is known as an *overall similarity* (OS) response. For separable stimuli, under full attention and unspeeded conditions, there is a strong preference for adults to group stimuli 7 and 8 together, i.e., the stimuli that are identical on one stimulus dimension but not the other. This is sometimes called a ‘dimensional’ response, but we prefer the less ambiguous term *identity* (ID) response (Wills et al., 2015). For separable stimuli, stimulus 7 and 8 are grouped together because the identity on one dimension overwhelms the fact that stimuli 6 and 8 are more similar overall.

An important thing to appreciate about the triad task is that while there is always one dimension on which two stimuli are identical, which dimension that is varies unpredictably from trial to trial. For example, while the first trial might involve stimulus triad 6-7-8 in Figure 1, the next might involve triad 1-3-7. In this case, the identity is on the saturation dimension, and an Identity response involves classifying stimuli 1 and 7 together. Thus, in order to classify on the basis of Identity, the participant must be sensitive to both stimulus dimensions, and weigh more heavily the dimension on which there is an identity.

In addition to overall similarity and identity responding, another possible response pattern in the triad task is that the participant’s classifications are made on the basis of a single dimension. In other words, a *unidimensional* (UD) strategy. For example, a participant might classify on the basis of stimulus brightness. In this case, they would classify stimuli 7 and 8 together in the triad 6-7-8, and stimuli 1 and 3 together in triad 1-3-7. Thus, the classification of any single triad supports at least two hypotheses about the participant’s behavior. For example, classifying 1 and 3 together from triad 1-2-3 supports both an Overall Similarity hypothesis, and a single-dimension (brightness) hypothesis. It is thus crucial that the participant’s responses are considered as a set across multiple trials, some trials involving brightness-identical triads and others saturation-identical triads. It was the lack of a full appreciation of this point that led to the over-extension of Differentiation Theory to separable stimuli, and the adoption of response-set analysis that resolved this issue (Raijmakers et al., 2004; Thompson, 1994; Wills et al., 2015).

In the current experiments, we apply response-set analysis of the triad task to the effects of stimulus presentation time on classification of integral stimuli varying in brightness and saturation. The decision to use stimulus presentation time in the current experiments was motivated by our previous use of this procedure in a comparable experiment with separable stimuli (Wills et al., 2015, Exp. 2, triad procedure with squares varying in size and brightness). Combination Theory predicts an increase in the prevalence of single-dimension classification with a decrease in stimulus presentation time. Differentiation Theory predicts the opposite.

The reduction of stimulus presentation time makes classification data more ‘noisy’ (Wills et al., 2015).^1^ In anticipation of this increased noise under short presentation times, we tested about twice as many participants in our short-stimulus-presentation-time condition as in our long-stimulus-presentation-time condition.

## EXPERIMENT 1

### Methods

#### Participants.

Forty-six psychology students from the University of Plymouth participated for partial course credit; the sample size was determined by the number of course credits available at the time of testing. Our experiment had sufficient (80%) power to detect medium-to-large effects (*w* = 0.40) in a chi-squared test. An effect size of approximately similar magnitude (*w* = 0.33) had previously been observed when applying a more subtle reduction in stimulus presentation time to squares varying in size and brightness (Experiment 2, Wills et al., 2015). Hence, the sample size provided an initial test of our hypothesis within the resources available. Power calculations were performed using the R package pwr (Champely, 2020). The University of Plymouth Faculty Ethics Committee approved all reported experiments.

#### Stimuli and Apparatus.

Eight colored squares, 8 mm on a side, were used (see Figure 1). The stimuli were of a red hue (Munsell 5R), and varied in chroma (4–12) and brightness (2–6); this is similar to the color space employed in Nosofsky (1987). Note that, in the Munsell system, two units of chroma are psychologically equivalent to one unit of brightness (Nickerson, 1936; Nosofsky, 1987; Shepard, 1958). The positioning of the stimuli within stimulus space followed our previous experiments (Milton et al., 2008; Wills et al., 2015). Eight stimulus triads were selected within this stimulus set (1-3-7, 1-5-7, 2-4-8, 2-6-8, 1-2-3, 1-2-4, 5-7-8, 6-7-8). There are six different ways in which three stimuli can be placed in three spatial locations (e.g., for the 1-3-7 triad, these would be 1-3-7, 1-7-3, 3-1-7, 3-7-1, 7-1-3, 7-3-1). The three stimuli in each triad were presented simultaneously in a horizontal line, with each square separated from the next by an edge-to-edge distance of 8 mm. Thus, each of the eight triads had six different instantiations, resulting in 48 physically different triads per experiment. Stimuli were presented on a 22-inch Philips LED monitor, against a mid-grey background, using E-prime 2; responses were collected using a standard PC keyboard. Participants sat approximately 50 cm from the screen; the whole stimulus triad thus subtended approximately five degrees of visual angle horizontally, and one degree vertically.

#### Procedure.

Participants were arbitrarily assigned to one of two conditions, short presentation time or long presentation time. The stimulus presentation times used were 100 ms and 2000 ms. At the beginning of each trial, the screen displayed the message “Ready?” and the participant pressed a key to continue. After this, a small fixation cross was presented in the center of the screen, for 500 ms. The stimulus triad was then presented for the appropriate duration, and then immediately replaced by the message “Odd one out?” The participant pressed the number key 1, 2 or 3 to indicate the left, middle, or right stimulus, respectively. The next trial began immediately upon detection of a response.

Each of the 48 physically different stimulus triads were presented twice, with order of presentation randomized for each participant. The randomization was constrained such that each block of eight trials contained exactly one of the eight logical triads (1-3-7, 1-5-7, etc.). At the end of each block, the participants received an on-screen reminder of the instructions, and pressed a key when they were ready to continue.

#### Strategy Analysis.

To determine the strategy used by each participant, the participants’ responses were compared to each of the three categorization strategies mentioned above: unidimensional (UD), overall similarity (OS), and identity (ID). Additionally, we checked to see whether any participants were best described by a Response Bias strategy (Bias). To determine the strategy used by each participant, we first determined for each of the 48 possible triad stimuli what the response should be given each of these strategies. For the UD strategy, the response would be the pair that was be closest on a particular dimension. For the OS strategy, the response would be the pair that was the most similar overall. For the ID strategy, the response would be the pair that shared an identical feature on either dimension. For the Bias strategy, the response would be where the participant pressed the same key throughout the experiment. We then counted, for each strategy and participant, how many of the participant’s responses matched the predicted response of that strategy. Then, the participant’s strategy was the strategy that best matched the participant’s responses.

### Results and Discussion

Table 1 shows that there was a strong preference for overall similarity classification at 2000 ms. This is expected from previous research and consistent with both theories. However, shortening the presentation time to 100 ms appeared to have little effect. One participant was best fit by a response-bias model (i.e., always pressing the same key), one by the assumption they were responding on the basis of a single dimension. All the other participants remained best fit by an overall similarity account. Bayesian analysis, conducted with the BayesFactor R package (Morey & Rouder, 2024), indicated that the ratio of unidimensional to overall similarity classifiers was unaffected by our manipulation, *BF*_10_ = 0.14. This result is not diagnostic between Combination Theory and Differentiation Theory. Differentiation Theory can attribute the lack of increase in overall-similarity classification to a ceiling effect, and Combination Theory can attribute it to the manipulation being insufficiently potent to produce a detectable effect.

**Table 1.** Number of participants (and proportion of participants) best fit by a unidimensional (UD), overall similarity (OS), identity (ID), or response bias (Bias) model, as a function of stimulus presentation time, in Experiment 1.

Time	UD	OS	ID	Bias
100 ms	1 (0.0345)	27 (0.931)	0	1 (0.0345)
2000 ms	0 (0)	17 (1.00)	0	0 (0)

In a further, exploratory, analysis we fitted the same response models to each eight-trial block of responses separately, deriving the number of blocks which, for each participant, were best fit by each model. Although this analysis is a novel one for the triad task, we have employed it in previous experiments using the match-to-standards categorisation procedure (Wills et al., 2013). We speculated that this analysis might be more sensitive to low levels of unidimensional classification, assuming that, due to both internal and external noise, participants sometimes successfully classified on the basis of both dimensions but other times did not have time to combine both dimensions and hence responded on the basis of a single dimension. This analysis was conducted on 45 participants, as one participant had been found to best fit a response-bias model. Response-bias models can only be fit at a whole-participant level, not an individual-block level, because it is only at the participant level that the physical position of the stimuli on the screen is counterbalanced.

On 13% of blocks, models tied for first place; these blocks were removed from further analysis. Table 2 shows the results of this by-block analysis. The crucial result is that participants in the 100 ms condition produce more classification blocks best fit by a unidimensional response model than participants in the 2000 ms condition, *BF*_10_ = 4.92. Hence reducing the stimulus presentation time increased unidimensional responding, even for these integral stimuli. This result is predicted by Combination Theory, and disconfirms Differentiation Theory.

**Table 2.** Mean proportion of unidimensional (UD), overall similarity (OS), and identity (ID) blocks, as a function of stimulus presentation time, in Experiment 1.

Time	UD	OS	ID
100 ms	0.119	0.881	0.000
2000 ms	0.018	0.975	0.007

## EXPERIMENT 2

A potential criticism of Experiment 1 is that it relies on a post-hoc analysis which, despite having precedents in the literature, was only engaged after our *a priori* analysis failed to reveal conclusive results. To address this potential criticism, we conducted a direct replication.

### Methods

Fifty psychology students from the University of Plymouth participated for partial course credit; this sample size has sufficient (84%) power to detect the medium-to-large effect observed in the by-blocks analysis of the previous experiment (*d* = 0.83). Power calculations were performed using the R package pwr (Champely, 2020). The stimuli, apparatus, and procedure, were identical to the previous experiment.

### Results and Discussion

One participant was excluded due to being best fit by a response-bias model. On 10% of blocks, models tied for first place; these blocks were removed from further analysis. Table 3 shows the key results, which are similar to the previous experiment. Crucially, shorter presentation times (compared to longer presentation times) once again increased the prevalence of unidimensional classification of these integral stimuli, *BF*_10_ = 1047. This Bayesian calculation makes use of a prior based on the size of the effect in the previous experiment. Specifically, following Dienes (2011), we used a normally-distributed prior of effect sizes, centered on the observed difference in the previous experiment, and with a standard deviation of half that observed difference. There is also Bayesian evidence for the effect of stimulus presentation time on unidimensional responding if one entirely ignores the prior provided by the previous experiment and uses a non-directional test against a non-informative prior, as performed in the previous experiment, *BF*_10_ = 3.50.

**Table 3.** Mean proportion of unidimensional (UD), overall similarity (OS), and identity (ID) blocks, as a function of stimulus presentation time, in Experiment 2.

Time	UD	OS	ID
100 ms	0.142	0.850	0.008
2000 ms	0.012	0.988	0.000

## EXPERIMENT 3

Our final experiment had two purposes. First, we wished to confirm that the stimuli as presented in the first two experiments met one standard Garner definition of integrality, i.e., the pairwise similarity ratings were better fit by a Euclidean than a city-block multidimensional scaling (MDS) solution (Garner, 1976). Color and brightness are only integral when the difference between colors is sufficiently small (Stalmeier & de Weert, 1991) so this was worth checking; although earlier work by Hyman and Well (1968), who performed MDS analysis for a stimulus set similar to the current one, seems to make such confirmation likely.

Second, we wished to assess the closeness of the resulting MDS solution to the solution provided by the Munsell color codes attributed to these stimuli. If differences were to be found, we would then use the MDS solution in a re-analysis of the triad data that represents the stimuli in terms of a psychological, rather than physical, stimulus space.

### Methods

#### Participants, Apparatus, and Stimuli.

Twenty-four participants were tested in this experiment; the sample size was determined by the number of course credits available at the time of testing, was similar to previous experiments of this type (e.g., Bergman et al., 2016; Gaissert & Wallraven, 2012; Livingston et al., 1998; Shin & Nosofsky, 1992) and is in line with the sample size shown by Rodgers (1991) to lead to good metric recovery in multidimensional scaling. PsychoPy software (Pierce, 2007), version 1.83, was used to present the stimuli and to collect responses via a standard PC keyboard and mouse. The stimuli were the same as in the two previous experiments.

#### Procedure.

**Figure 2.**
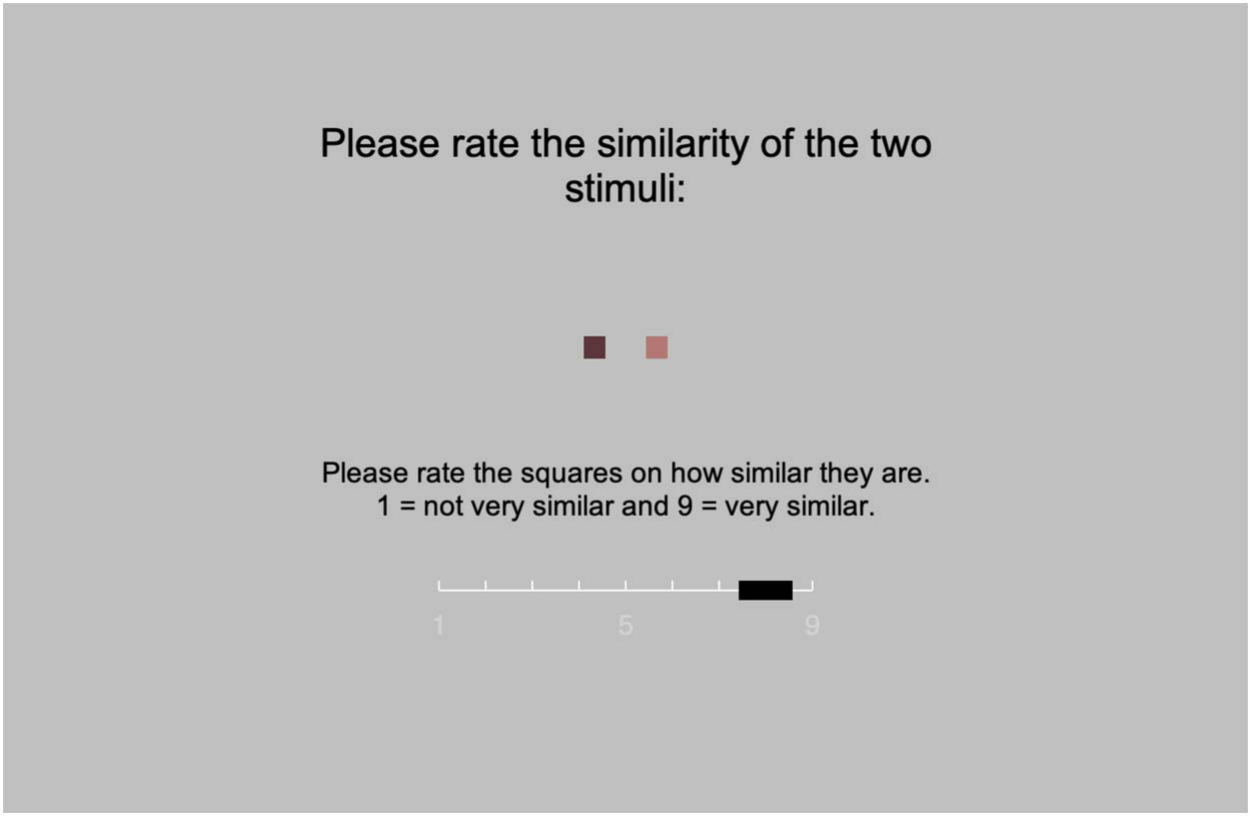
A screenshot of a single example trial from Experiment 3.

After some initial instructions explaining the task, the experiment began. On each trial, two square stimuli were shown in the center of the screen, arranged horizontally, and placed 2 cm apart as measured from their centers (see Figure 2). Participants were asked to rate the similarity of each pair of stimuli using a 1–9 scale. The scale was visually presented on the screen, below the square stimuli, along with text specifying that “1 = not very similar” and “9 = very similar”. The number 5 was also indicated on the scale, but not further labelled. A moveable rectangular slider was present on this scale. Initially placed above the number 5, participants moved this slider to one of the nine available ratings using the mouse and indicated their response with a mouse click. The screen cleared immediately after the participant’s response, and the next trial began one second later.

The experiment had two blocks of 56 trials. A block comprised two presentations of each possible pair of the eight stimuli, with left-right position of the stimulus pair counterbalanced across those two presentations. Trial ordering was randomized separately for each block and participant.

### Results and Discussion

The average pairwise similarity ratings for the eight stimuli are shown in Table 4. Two-dimensional, non-metric multidimensional scaling (Kruskal, 1964) was applied to these data, using the isoMDS function of the R package MASS (Ripley & Venables, 2025). The stress of the Euclidean solution (0.04) was lower than the city-block solution (3.19), implying that these stimuli are better considered as integral than separable by Garner’s operational definition.

**Table 4.** Pairwise similarity ratings.

	1	2	3	4	5	6	7
2	4.32						
3	7.68	4.54					
4	5.03	7.12	6.38				
5	4.47	3.07	5.90	4.28			
6	3.78	3.33	4.79	4.65	6.60		
7	3.81	2.36	4.71	3.27	7.66	5.66	
8	3.57	2.97	3.92	3.95	5.61	7.34	5.33

**Figure 3.**
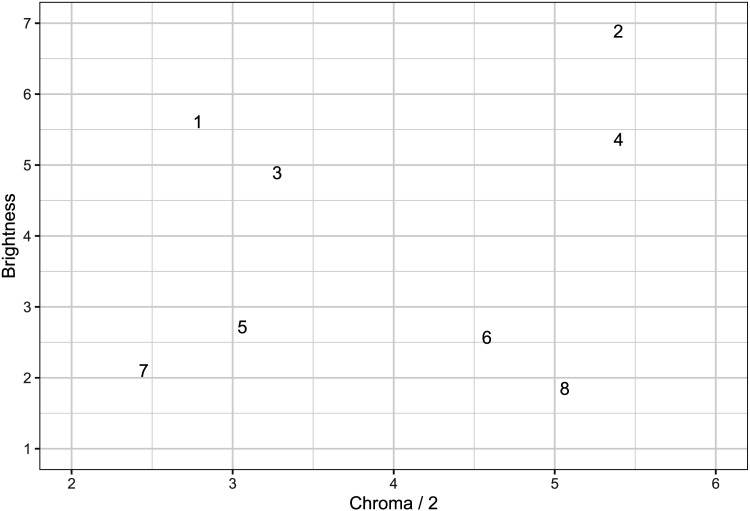
Multidimensional scaling solution.

Figure 3 shows the Euclidean MDS solution, scaled and Procrustean rotated for best fit to the co-ordinates of the stimuli in the Munsell color system; these rigid transformations do not affect the distance relationships in a Euclidean MDS solution. The procrustes function of the R package vegan (Oksanen et al., 2025) was used for this part of the analysis. Following standard practice, we assumed that, in the Munsell system, two units of chroma are psychologically equivalent to one unit of brightness (Nickerson, 1936; Nosofsky, 1987; Shepard, 1958).

Inspection of Figure 3 indicates that, for six of the eight stimuli, the MDS solution shows similarity relations comparable to those in the Munsell co-ordinates. The exceptions are stimuli 2 and 4, which would appear to have been perceived as somewhat brighter and more saturated than their Munsell co-ordinates would indicate. This may have been a function of our use of commodity hardware for screen display.

Given these moderate discrepancies between the Munsell co-ordinates and the multidimensional scaling solution, we re-analyzed the data from the previous two experiments, combining the two datasets and using the MDS co-ordinates as the inputs to our response models, rather than the Munsell co-ordinates.

Two participants were excluded due to being best fit by a response-bias model. On 25% of blocks, models tied for first place; these blocks were removed from further analysis. Table 5 shows the results of our re-analysis. Using the MDS co-ordinates for these stimuli approximately doubled the magnitude of the effect observed with the Munsell co-ordinates; unidimensional responding rose from 9% at 2000 ms to 32% at 100 ms. Bayesian analysis, employing a non-informative prior, provides very strong support for an effect of stimulus presentation time on the prevalence of unidimensional responding, *BF* = 892. Hence, overall, the three experiments presented in the current paper provide strong evidence for the effect predicted by Combination Theory, and disconfirm Differentiation Theory.

**Table 5.** Mean proportion of unidimensional (UD), overall similarity (OS), and identity (ID) blocks, as a function of stimulus presentation time.

Time	UD	OS	ID
100 ms	0.319	0.681	0
2000 ms	0.086	0.914	0

Further inspection of Figure 3 reveals that no stimulus is identically placed on either dimension (this is true even for stimuli 2 and 4 on the chroma dimension). As a consequence, the Identity response model can never predict participants’ responses, leading to reported zero prevalence of Identity responding in Table 5. It would in principle be possible to generalize the Identity response model such that it could deal with near-identity (such as stimuli 2 and 4 on chroma) effectively, and the work of Smith (1989) suggests a way in which this could be done. However, given the very low prevalence of ID classification observed for these stimuli in our earlier analysis, where the use of Munsell co-ordinates would have made such responding detectable if it had occurred, such a generalization of the Identity model would be unlikely to change the conclusions of the current work.

### Additional Analyses

**Figure 4.**
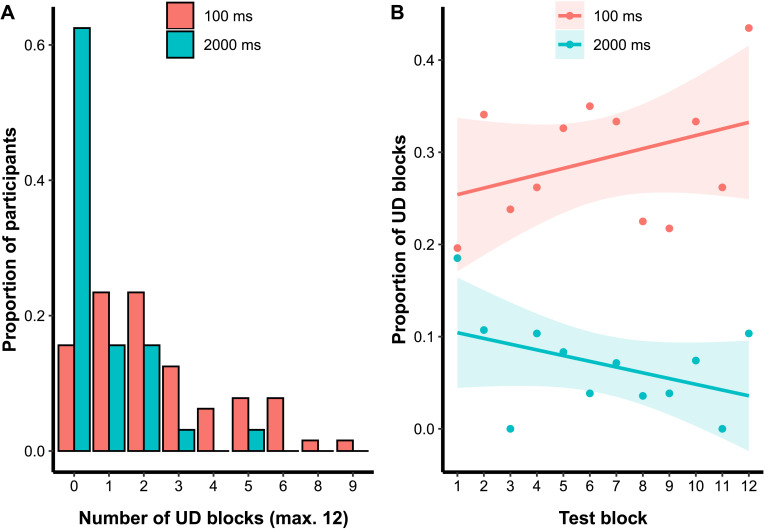
(A) Distribution of unidimensional (UD) responding across participants. (B) Prevalence of UD responding across test blocks.

It is reasonable to ask how the proportion of UD responding is distributed—across participants, across the blocks of the experiment, and across the stimulus space. Starting with participants, Figure 4A shows, for each stimulus presentation time, the distribution of UD prevalence across participants. At 2000 ms, the majority of participants have no blocks that are best fit by the UD model, and the distribution drops off rapidly as the number of UD blocks increases. At 100 ms, the distribution is broader and shifted to the right, with the modal number of UD blocks being between 1 and 2. Thus, the effect of shortening presentation time is best described as having a small but reliable effect across participants, rather than a large effect for a few participants.

Turning to how the proportion of UD responding evolves over the 12 test blocks, Figure 4B shows a numerical trend for UD responding to increase at 100 ms and decrease at 2000 ms. However, in a linear regression model with proportion of UD blocks as the dependent variable, and test block and presentation time as the independent variables, only the main effect of presentation time is significant, *t*(20) = −2.61, *p* = 0.02, with the main effect of block non-significant, *t*(20) = 1.42, *p* = 0.17, and the interaction of these two terms marginal, *t*(20) = −1.88, *p* = 0.07. Thus, there is no strong evidence that the proportion of UD responding evolves across the experiment, and the analysis is consistent with the idea that performance is relatively stable across this short experiment. However, if anything, the size of the effect seems to grow as the experiment proceeds.

The question of how the effect is distributed across stimulus space is harder to answer. To see why this is, first recall that every trial involves the presentation of three stimuli. On some trials, this triad will span the whole of the brightness dimension (e.g., 1-3-7). On others, it will span the whole of the chroma dimension (e.g., 1-2-3). This might lead one to the conclusion that one could conduct four sub-analyses—low chroma (spanning brightness), high chroma (spanning brightness), low brightness (spanning chroma), and high brightness (spanning chroma). However, each of these sub-areas of the stimulus space are, taken separately, non-diagnostic of OS versus UD responding. For example, low chroma, spanning brightness, contains two triads: 1-3-7 and 1-5-7. In both cases, the OS-consistent response (grouping 1 & 3, grouping 5 & 7, respectively) is also a UD-consistent response (brightness UD in both cases). Thus, it is crucial to have both chroma-spanning and brightness-spanning triads in the analysis.

This in turn means that any analysis of these data must include at minimum 75% of the stimulus space. Nonetheless, one can run four overlapping sub-analyses, each removing triads containing stimuli in one quadrant of space (e.g., 6 & 8, the HCLB—high chroma, low brightness—condition). If there is *evidence for the absence* of an effect (of presentation time on UD sorting) after removing one quadrant, that quadrant was critical in producing the effect. However, this was not the pattern of results observed. Instead, for three of the four quadrants, there remained strong evidence for the effect after the quadrant’s removal, *BF* = 188800, 406 and 125 for LCHB, HCHB, and HCLB quadrant removal, respectively. For the fourth quadrant (LCLB—low chroma, low brightness) the Bayesian evidence was inconclusive but still ancedotally in favor of an effect, *BF* = 1.39. We thus conclude there is no clear evidence that any one quadrant is crucial to the effect, although if one nevertheless wanted to single out a quadrant for importance, it would probably be LCLB.

In summary, and to a first approximation, these additional analyses show that the effect of reducing presentation time is distributed roughly evenly across participants, across the experiment, and across the stimulus space. Nonetheless, there are some hints that the effect may get stronger the longer one tests, and as the stimuli reduce in chroma and brightness.

## GENERAL DISCUSSION

As stimulus presentation time decreases, the prevalence of single-dimension classification of multi-attribute stimuli increases (Wills et al., 2015). This phenomenon is well established for separable stimuli (e.g., squares varying in size and brightness), but the current experiments are the first demonstration for integral stimuli (specifically, for squares varying in saturation and brightness). Intuitively, this effect of stimulus presentation time on integral stimuli may seem surprising. After all, under conditions of unlimited stimulus presentation time, it is well established that selectively attending to integral stimulus dimensions requires effort (Garner, 1976). Such observations have led some to conclude that cognition begins with an undifferentiated stimulus whole, which is analyzed into its components only with effort (Lockhead, 1972 et seq.); a view subsequently described as Differentiation Theory (Wills et al., 2015). However, our results support an approximately opposite account—cognition begins with stimulus attributes, which are combined if time and mental resources allow (Neisser, 1967 et seq.); an account subsequently described as Combination Theory (Wills et al., 2015). Once attributes are combined, selective attention is somewhat effortful, and that effort is greater for integral than for separable stimuli. Under a Combination Theory account, synthesis—rather than analysis—is the more appropriate chemical metaphor. Integral stimuli, rather than being slowly analyzed, are quickly synthesized.

Indeed, this distinction may at least partially explain the virtual absence of ID sorting that we observed with integral stimuli in the long presentation time condition, in contrast to the relatively high levels of ID sorting with separable stimuli previously found under a similar time constraint (e.g., Wills et al., 2015). For separable stimuli, the ability to selectively attend and switch between dimensions is assumed to become easier as presentation time increases, which makes ID responding an easier and more commonly used approach. However, for integral stimuli, once synthesis has occurred, it is much harder to selectively attend to the individual dimensions than for separable stimuli and harder still to switch between the two dimensions, which is necessary to consistently make ID responses. This added difficulty of selectively attending to integral than separable dimensions after they have been combined may therefore explain the virtual absence of ID sorting in the current experiments, in contrast to the relatively high prevalence of this approach with separable stimuli.

The current experiments concern robust but subtle effects at short presentation times. One might ask, if Combination Theory is accurate, why the effects are not larger? Combination Theory states that integral dimensions are initially represented separately, but that integral dimensions are rapidly combined, and, once combined, are hard to selectively attend. Thus, observing any unidimensional classification of integral stimuli will likely depend on having very short stimulus presentation times, and a highly sensitive analysis method. The theoretical point is that the direction of the effect (increased unidimensional responding with decreased stimulus presentation time) is as predicted by Combination Theory, and counter to the predictions of Differentiation Theory.

A range of other evidence is more consistent with Combination Theory than Differentiation Theory. In the case of separable dimensions, there is evidence consistent with Lamberts’ EGCM model of the time course of categorisation, which assumes separable stimulus dimensions are sampled separately and combined over time (Lamberts, 1995, 1998, 2000) and evidence consistent with logical-rule models of classification, which assume decisions about separable dimensions are made independently of each other (Fific et al., 2008, 2010; Little et al., 2011; Moneer et al., 2016). Eye tracking data (Rehder & Hoffman, 2005) is also supportive of Combination Theory in the context of the classification of separable stimuli. Also of interest is work on the speeded classification of faces. Faces are generally considered to be processed holistically. However, a range of research now converges on the conclusion that holistic processing of faces emerges relatively late in the time course (Fitousi, 2019; Goshen-Gottstein & Ganel, 2000; Macrae & Martin, 2007), at least 400 ms after stimulus onset (Lynch et al., 2021).

In summary, our results add to the growing body of evidence that supports Combination theory over Differentiation theory for both separable and integral stimuli. It is nonetheless worth emphasizing that our results do not, of course, challenge the existence of Garner’s classic integral-separable distinction—Combination and Differentiation Theory both make use of this distinction, just in different ways. It is also worth noting that whilst our pattern of results appear to be robust, the precise mechanisms that underlie the effect remain uncertain and Combination Theory does not make any direct predictions in this regard.

Although the current experiments concern the presentation of simple stimuli in the laboratory, the distinction between Differentiation and Combination Theory is of broader relevance. The terms ‘analytic’ and ‘nonanalytic’ (or ‘holistic’) are broadly applied in psychology as theories of modes of thought. Such terms seem to assume the correctness of Differentiation Theory (otherwise terms such as ‘synthetic’ would be more appropriate). Given that Differentiation Theory seems to be largely the wrong theory for the classification of simple stimuli, an investigation of the extent to which the predictions of Differentiation Theory are correct across psychology more generally seems worthy of further examination. For example, a form of Differentiation Theory seems to underlie the proposal that the training of radiologists could be improved by distracting them with a secondary task (Filoteo et al., 2010), while Combination Theory predicts that this is likely to be harmful (Newell et al., 2013). A second example—one of the key ways WEIRD (Western Educated Industrialized Rich and Democratic) populations are described as differing from some other cultures is in the unusual extent to which WEIRD thought is characteristically ‘analytic’ rather than ‘holisitic’ (Henrich et al., 2010). Such a description presupposes a form of Differentiation Theory.

Returning to the experiments reported in the current paper, the same-lab replication of our results reduces the chances that we are reporting a false positive, but replication by an independent lab would further increase confidence, and we are keen to support such efforts. Our materials and analysis methods are publicly available. One crucial aspect for successful replication of our results is the well-controlled presentation of precisely-defined stimuli; the logic of the experiment requires, for example, that the location of stimuli in physical stimulus space (Figure 1) corresponds reasonably closely to their position in psychological stimulus space. Investigations of our own stimuli (reported in Experiment 3) support the conclusion that, in our case, the correspondence was sufficiently close that our conclusions remain valid (in fact, use of a psychological stimulus space strengthens the support for our conclusions). In a similar vein, it is possible that our unmasked stimulus presentation meant that participants perceived the stimuli for longer than 100ms, due to visual persistence (Sperling, 1960). Future investigators may wish to further tighten experimental control through the use of a post-stimulus mask. However, it seems unlikely that this would reverse the direction of the effect of presentation time, and it is the direction that is the subject of the hypotheses under test. To further generalise our results, it may be helpful to consider other integral stimulus sets, perhaps with pure tones differing in pitch and loudness (Grau & Nelson, 1988) or sets with more than two dimensions (e.g., Nosofsky & Palmeri, 1996; Vigo, Doan, & Zhao, 2022).

It may also be worth noting that, given our effective stimulus presentation time of at least 100 ms (see above), there is ample time for the very earliest stages of visual processing of our stimuli to complete—time to peak response is approximately 60 ms for foveal cones (Masland, 2017), for example. Nonetheless, psychophysics and visual neuroscience are relevant to discussion of Combination Theory versus Differentiation Theory. For example, evidence suggests that luminance is represented at least somewhat separately from hue in the lateral geniculate nucleus (Ghodrati et al., 2017), and later combined through recurrent inhibitory activity in early visual cortex (Xing et al., 2015). The current experiments support the idea that, early in the time course of perception, luminance and hue are available separately to drive behavior. The later combination of stimulus components that are initially represented separately seems more consistent with Combination Theory than Differentiation Theory.

It would also be interesting to see which formal process models of classification can accommodate the current results. EGCM (Lamberts, 1995) seems a likely candidate, as does any other model that explicitly combines stimulus dimensions over time (Cohen & Nosofsky, 2003). A further promising approach is Vigo, Wimsatt, et al. (2022) recent dual discrimination invariance model (DDIM) which has been shown to provide an excellent account of the classification of three-dimensional integral stimuli, although it may need to be extended to include a time component to provide a full explanation of our current data (Vigo, Doan, & Zhao, 2022). In contrast, theories such as COVIS (Ashby et al., 1998), in which responding on the basis of a single dimension is a function of the effortful use of the rule-based system, seem conceptually closer to Differentiation Theory, and hence may require some modification in order to accommodate the present results.

Finally, it is reasonable to ask whether there are any points in the perceptual time course, for any type of stimulus, for which the evidence is more consistent with Differentiation Theory than Combination Theory. In our twenty-year investigation of this topic, starting with Milton and Wills (2004), across different stimuli and procedures, including comparative (Wills et al., 2009) and neuroscience (Milton et al., 2009) methods, we have often looked for such evidence, but only found it once. In Experiment 4 of Milton et al. (2008), using a match-to-standard procedure and schematic boat stimuli, as presentation time reduced from 384 ms to 256 ms, the proportion of UD sorts reduced and the proportion of OS sorts increased. This result, which is problematic for Combination Theory, was opposite to that seen at both smaller (64–256 ms) and longer (384–4096 ms) time courses. Given the growing evidential basis for Combination Theory, it may be worth revisiting this effect.

## ACKNOWLEDGMENTS

The authors thank Anna Robertson and Gemma Williams for their assistance in data collection. The experiments in this manuscript were approved by Faculty of Health and Human Sciences Research Ethics Committee, University of Plymouth. Reference Number: 14/15-444.

## AUTHOR CONTRIBUTIONS

C.E.R.E.: Conceptualization; Data curation; Formal analysis; Methodology; Validation; Visualization; Writing – review & editing. F.M.: Conceptualization; Methodology; Validation; Writing – review & editing. A.J.W.: Conceptualization; Data curation; Formal analysis; Investigation; Methodology; Project administration; Resources; Software; Supervision; Validation; Visualization; Writing – original draft; Writing – review & editing.

## DATA AVAILABILITY STATEMENT

Stimulus files, raw data, and a reproducible manuscript including all analysis code, will be available on manuscript acceptance at https://github.com/ajwills72/pu084github.

## Note

^1^ Here, ‘noisy’ means being either more erratic in the application of a strategy, or more inconsistent in the selection of a strategy in the first place. Wills et al. measured this using the proxy of the number of responses that fit the winning strategy.
